# Inhibition of cancer cell proliferation by midazolam by targeting transient receptor potential melastatin 7

**DOI:** 10.3892/ol.2013.1129

**Published:** 2013-01-11

**Authors:** YUNLING DOU, YUAN LI, JINGKAO CHEN, SIHAN WU, XIAO XIAO, SHANSHAN XIE, LIPENG TANG, MIN YAN, YOUQIONG WANG, JUN LIN, WENBO ZHU, GUANGMEI YAN

**Affiliations:** 1 Department of Pharmacology, Zhongshan School of Medicine, The First Affiliated Hospital of Sun Yat-Sen University, Guangzhou 510080, P.R. China; 2 Department of Anesthesiology, The First Affiliated Hospital of Sun Yat-Sen University, Guangzhou 510080, P.R. China;; 3 Department of Anesthesiology, State University of New York Downstate Medical Center, Brooklyn, NY 11203, USA

**Keywords:** transient receptor potential melastatin 7, midazolam, proliferation, cell cycle arrest, human head and neck carcinoma

## Abstract

Transient receptor potential melastatin 7 (TRPM7), a Ca^2+^-permeable channel, has been demonstrated to be present in cancer cells and involved in their growth and proliferation. The present study used midazolam, a benzodiazepine class anesthesic, to pharmacologically intervene in the expression of TRPM7 and to inhibit cancer cell proliferation. Midazolam significantly inhibited the growth and proliferation of FaDu human hypopharyngeal squamous cell carcinoma cells, concurring with the induction of G_0_/G_1_ cell cycle arrest and blockage of Rb activation. Central-type and peripheral-type benzodiazepine receptor antagonists did not abrogate proliferation inhibition by midazolam, while the specific TRPM7 agonist bradykinin reversed this effect. In addition, other benzodiazepines, diazepam and clonazepam also exhibited anti-proliferative activities. The inhibitory activity on cancer cell growth and proliferation, combined with the TRPM-dependent mechanism, reveals the anticancer potential of midazolam as a TRPM7 inhibitor and supports the suggestion that TRPM7 is a valuable target for pharmaceutical intervention.

## Introduction

Ca^2+^, a ubiquitous signal ion, controls a series of physiological processes, including cell proliferation, metabolism and gene transcription. Ca^2+^ signalling is essential for all eukaryote cells, including cancer cells, to grow and proliferate (1). Altered expression of specific Ca^2+^ channels and pumps causes over-sufficiency in growth signals, promoting cancer cell proliferation (2). Studies evaluating the ability of Ca^2+^ to regulate cell death and proliferation present an opportunity for a new set of drug targets in cancer (2,3).

Tumor cells are non-excitable cells with few voltage-gated Ca^2+^ channels, among which the transient receptor potential (TRP) channels have been recognized as the main Ca^2+^ entry pathway (4). Transient receptor potential melastatin 7 (TRPM7), one member of the TRPM channel subfamily of TRP channels, has been shown to be present in human head and neck squamous carcinoma FaDu and SCC25 cells. Suppression of TRPM7 expression or blockage of TRPM7 currents leads to inhibition of the growth and proliferation of FaDu and SCC25 cells (5), which may provide an opportunity for therapeutic intervention.

In the present study, the levels of TRPM7 were pharmacologically manipulated, in order to use its downregulation as a tool to repress the cell proliferation of FaDu cells. The aim was to test whether midazolam [molecular weight 325.77, a clinically widely-used benzodiazepine (BZ) anesthetic] inhibits cell growth and proliferation by repressing TRPM7 expression in FaDu cells. We also aimed to determine whether this effect was unique to midazolam or common to benzodiazepines. We propose that the present results, showing the proliferation-inhibitory activity of midazolam, not only lay a theoretical foundation for the preferential use of midazolam as the anesthetic during tumorectomy, but also identify TRPM7 as a therapeutic target for cancer.

## Materials and methods

### Antibodies and reagents

Midazolam, diazepam, clonazepam and flumazenil were purchased from Nhwa Pharmaceutical Group (Jiangsu, China). PK11195 was obtained from Sigma, (St. Louis, MO, USA). Antibodies against cyclin D1, cyclin E, P21, P27, Rb and phosphorylated Rb were obtained from Cell Signaling Technology (1:1,000; Beverly, MA, USA), while tubulin antibody (1:5,000) was obtained from Sigma and CDK 2, 4 and 6 antibodies were purchased from Santa Cruz Biotechnology (1:500; Santa Cruz, CA, USA).

### Cell culture

FaDu human hypopharyngeal squamous cell carcinoma cells (ATCC HTB-43), were maintained in Eagle’s MEM with 10% fetal bovine serum (FBS; Invitrogen, Grand Island, NY, USA), 50 U/ml penicillin and 50 *μ*g/ml streptomycin. Cells were cultured in a 5% CO_2_ humidified atmosphere at 37°C. The study was approved by the Ethics Committee of Sun Yat-sen University, Guangzhou, China.

### Cell viability assay

The cell viability assay was performed with MTT (Sigma). Cells were seeded in 96-well plates and the initial cell number was adjusted to 3,000/well. Following drug treatment, 20 *μ*l MTT (5 mg/ml in PBS) was added to the medium to induce the production of formazan crystals. After 4 h, the MTT solution was aspirated off and 100 *μ*l dimethyl sulfoxide (Sigma) was added to solubilize the formazan crystals. The optical density (OD) was determined at 570 nm using an iMark™ Microplate Reader (Bio-Rad, Richmond, CA, USA). The cell viability rate = OD_treatment_ / OD_control_ (vehicle) × 100.

### Cell proliferation assay

For the cell proliferation assay, a cell proliferation ELISA kit for the thymidine analog 5-bromo-2′-deoxyuridine (BrdU; Roche Diagnostics, Mannheim, Germany) was used as per the manufacturer’s instructions. In brief, cells were seeded in 96-well plates and the initial cell number was adjusted to 3,000/well. Following drug treatment, the cells were labeled with BrdU for 4 h. Subsequently, anti-BrdU-POD Fab fragments and substrate were added to the medium. The optical density (OD) was determined at 405 nm using an iMark Microplate Reader. The results were normalized to the control (the group treated with vehicle).

### Cell death assay

Cell death was evaluated using a lactate dehydro genase (LDH) release assay. LDH release was quantified with a CytoTox 96 non-radioactive cytotoxicity assay kit (Promega, Madison, WI, USA) according to the manufacturer’s instructions. Cells were seeded in 96-well plates and the initial cell number was adjusted to 3,000/well. Following drug treatment, 50 *μ*l medium/well was transferred to another 96-well plate. The solution of LDH substrate (50 *μ*l) was added to the medium and incubated for 30 min. Subsequently, 50 *μ*l stop solution was added to stop the reaction and the absorbance was measured at 490 nm with an iMark Microplate Reader. The results were normalized to the control (the group treated with vehicle).

### Cell cycle analysis

After 24 h of serum starvation, the cells were exposed to the complete medium with 10% FBS. Following treatment, the cells were harvested by trypsinization, washed twice with cool PBS and fixed in 75% ethanol overnight at 4°C. Subsequently, the cells were incubated in solution with 50 mg/ml DNA-binding dye PI, 4 kU/ml RNase, 0.3 mg/ml NaF and 1 mg/ml sodium citrate for 30 min at 37°C away from light. Finally, the red fluorescence from the 488 mm laser-excited PI in every cell was analyzed with an EPICS ALTRA flow cytometer (Beckman Coulter, Fullerton, CA, USA) using a peak fluorescence gate to discriminate aggregates. The percentages of cells in the G_0_/G_1_, S and G_2_/M phases were determined from DNA content histograms using Multicycle for Windows (Phoenix Flow Systems, San Diego, CA, USA).

### Western blot analysis

Western blot analysis was performed as described previously (6). In brief, cells were scraped and then resuspended in protein extraction reagent. The cell lysate was centrifuged at 140,000 g for 10 min at 4°C and the supernatant was collected for electrophoresis. Prior to electrophoresis, the concentration of protein was determined using a BCA protein assay kit (Pierce, Rockford, IL, USA) following the manufacturer’s instructions. Equal amounts of proteins (30 *μ*g) were separated by 12% SDS-PAGE. After electrophoresis, the proteins were transferred to PVDF membranes, blocked with 5% skimmed milk in TBS for 2 h and reacted with antibodies overnight. After reaction with horseradish peroxidase-labeled secondary antibody, the immune complexes were visualized using the ECL-detection reagents according to the manufacturer’s instructions.

### Quantitative real-time PCR (qPCR)

Total RNA was extracted with TRIzol reagent (Invitrogen, Carlsbad, CA, USA) according to the manufacturer’s instructions. The purity and integrity of all isolated RNA samples was analyzed using agarose gel electro phoresis. The first strand of the cDNA was synthesized using SuperScript III reverse transcriptase (Invitrogen) with an oligo(dt) primer. The sequences of the PCR primers used were as follows: TRPM7, 5′-TGC AGC AGA GCC CGA TAT TAT-3′ (sense primer) and 5′-CTC TAT CCC ATG CCA ATG TAA GG-3′ (antisense primer); GAPDH, 5′-TCA CCA TCT TCC AGG AGC GAG A-3′ (sense primer) and 5′-ATG AGC CCT TCC ACG ATG C-3′ (antisense primer). qPCR was performed with Platinum SYBR-Green qPCR SuperMix-UDG (Invitrogen) and detected with a LightCycler 480 (Roche, Basel, Switzerland). The comparative CT method (2^−ΔΔCT^) was used to evaluate the relative quantities.

### Statistical analysis

Data are presented as the mean ± standard deviation (SD) of at least three separate experiments. The statistical significance was determined by ANOVA analysis. P<0.05 was considered to indicate a statistically significant difference.

## Results

### Midazolam inhibits the growth and proliferation of FaDu cells

**Figure 1:**
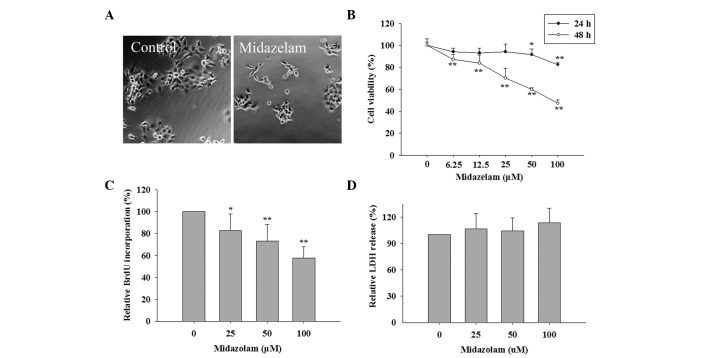
Midazolam inhibited the growth and proliferation of FaDu cells. (A) Phase-contrast image of cells treated with 0 (control) and 100 *μ*M mida zolam for 48 h (original magnification, ×200). (B) Dose- and time-dependent effect of midazolam on cell viability. FaDu cells were incubated with 0, 6.25, 12.5, 25, 50 and 100 *μ*M midazolam for 24 and 48 h. The data are the mean ± SD (n=5), ^**^P<0.01 compared with the control. Effects of midazolam on (C) cell proliferation by BrdU incorporation assay and (D) cell death by lactate dehydrogenase (LDH) release assay. FaDu cells were incubated with 25, 50 and 100 *μ*M midazolam for 48 h. The data are the mean ± SD (n=3), ^*^P<0.05, ^**^P<0.01, compared with the control.

To examine whether midazolam affects the growth of human hypopharyngeal squamous cell carcinoma FaDu cells, the changes in morphology and cell number of FaDu cells treated with 100 *μ*M midazolam were observed by phase-contrast microscopy. As shown in Fig. 1A, the bodies of the majority of cells exposed to midazolam appeared to be smaller than the controls and the cell number was significantly lower, indicating that midazolam inhibits the growth of FaDu cells. The MTT assay used to measure the relative counts of live cells showed that midazolam treatment for 24 h decreased the cell viability which was evident at 50 and 100 *μ*M. By 48 h, the cell viability had decreased at 6.25 *μ*M and been reduced to 47.1% at 100 *μ*M (Fig. 1B). To further investigate whether cell viability loss by midazolam was due to proliferative inhibition or cell death, the relative levels of BrdU incorpration representing cell proliferation and LDH release representing cell death were measured in FaDu cells treated with 25, 50 and 100 *μ*M midazolam. The data from Fig. 1C and D showed that midazolam dose-dependently reduced BrdU incorpration but did not trigger LDH release. Therefore, midazolam induced cell viability loss by inhibiting cell proliferation in FaDu cells.

### Midazolam triggers G_0_/G_1_ cell cycle arrest by regulating cell cycle regulators

**Figure 2:**
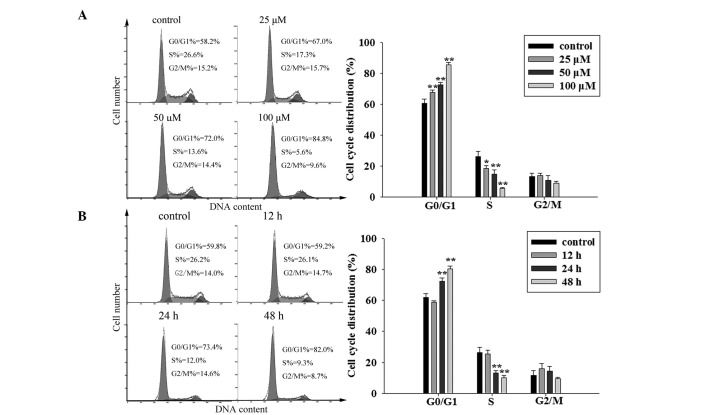
Midazolam induced G_0_/G_1_ cell cycle arrest in FaDu cells. (A) Dose-dependent effect of midazolam on cell cycle distribution. FaDu cells were treated with 0 (control), 25, 50 and 100 *μ*M midazolam for 48 h. (B) Time-dependent effect of midazolam on cell cycle distribution. FaDu cells were treated with 50 *μ*M midazolam for 0 (control), 12, 24 and 48 h. Representative cell cycle distribution (left) and statistical graph (right; n=3), ^*^P<0.05, ^**^P<0.01, compared with the control.

The cell cycle distribution determines the rate of cell proliferation. Generally, the percentage of cells in the S phase reflects the quantity of proliferating cells. Cancer cells are abnormal cells which have lost their balance of proliferation and apoptosis so the percentage in S phase is much larger than that of normal cells from the same tissues or organs (7). The data from three independent cell cycle analyses showed that midazolam reduced the mean S phase percentage from 26.1 to 18.4, 15.0 and 5.7% at concentrations of 25, 50 and 100 *μ*M, respectively. The mean G_0_/G_1_ phase percentage climbed from 60.5 to 67.7, 72.5 and 85.3% (at 25, 50 and 100 *μ*M midazolam, respectively), while no statistically significant differences were observed in the mean M phase percentages (Fig. 2A). In addition, the cell cycle distributions of FaDu cells in response to midazolam treatment for 12, 24 and 48 h were analyzed. The results showed that G_0_/G_1_ phase arrest induced by midazolam had begun by 24 h and was greater by 48 h (Fig. 2B). These data suggest that midazolam hinders the cells’ progression from G_1_ to S phase and that the checkpoint of the G_1_/S phase transition may be affected by midazolam.

**Figure 3:**
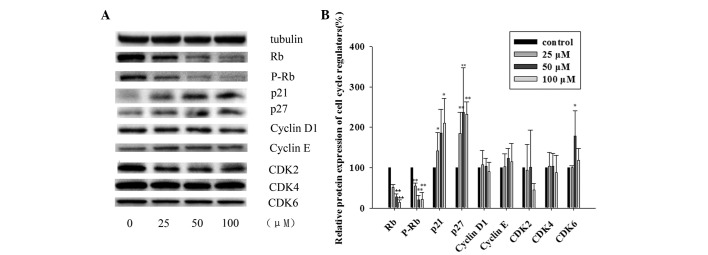
Midazolam prevented Rb activation by affecting the expression of cell cycle regulators. (A) Effect of midazolam on protein levels of Rb, phosphorylated Rb (p-Rb), p21, p27, cyclin D1, cyclin E, CDK2, CDK4 and CDK6. (B) Statistical graph of three independent experiments. Gray scales of proteins were normalized to housekeeping genes (tubulin, β-actin and GAPDH) and data are presented as values relative to the control. FaDu cells were treated with 0 (control), 25, 50 and 100 *μ*M midazolam for 48 h and then subjected to western blot analysis. The data are the mean ± SD (n=3), ^*^P<0.05, ^**^P<0.01, compared with the control.

The checkpoint is composed of cyclin D1 and E, cyclin-dependent kinase (CDK) 2, 4 and 6 (cyclin/CDK complex), p21 and p27 (CDK inhibitors), and Rb (a determinant of E2F1 release). Western blot analysis revealed that p21 and p27 proteins were significantly upregulated, while the total and phosphorylated Rb (active type) were markedly decreased, although the other proteins did not noticeably change (Fig 3). The data indicate that the reduction of active Rb is the main contributor to G_0_/G_1_ cell cycle arrest by midazolam.

### Proliferation-inhibitory effect of midazolam is benzodiazepine receptor (BR)-independent but TRPM7-dependent

**Figure 4:**
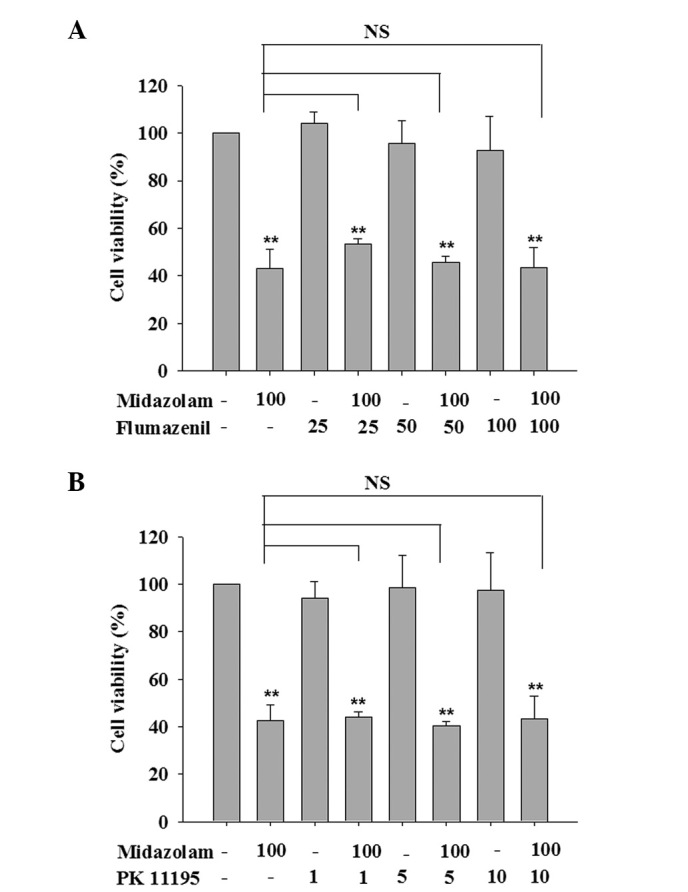
(A) CBR antagonist flumazenil and (B) PBR antagonist PK11195 did not abrogate midazolam-induced cell viability loss. FaDu cells were treated with 50 *μ*M midazolam, co-cultured with flumazenil (25, 50 and 100 *μ*M) or PK11195 (1, 5 and 10 *μ*M) for 48 h and then subjected to an MTT assay. The data are the mean ± SD (n=3), ^**^P<0.01, compared with the control (untreated group). CBR, central-type benzodiazepine receptor; PBR, peripheral-type benzodiazepine receptor.

The nervous system-inhibitory activity of midazolam is known to be mediated by BRs, including central-type BZ receptor (CBR) and peripheral-type benzodiazepine receptor (PBR). To determine the mechanism underlying the proliferation-inhibitory effect of midazolam, the role of CBR and PBR in this non-nervous system activity had to be clarified. Thus, the specific CBR antagonist flumazenil and PBR antagonist PK11195 were used to compete with midazolam to bind to CBR or PBR. At a concentration range within which cell viability was not affected, flumazenil (25–100 *μ*M) and PK11195 (1–10 *μ*M) were unable to reverse the proliferation loss induced by midazolam (Fig. 4). This result suggests that the mechanism underlying midazolam-induced FaDu cell proliferation is BR-independent.

**Figure 5:**
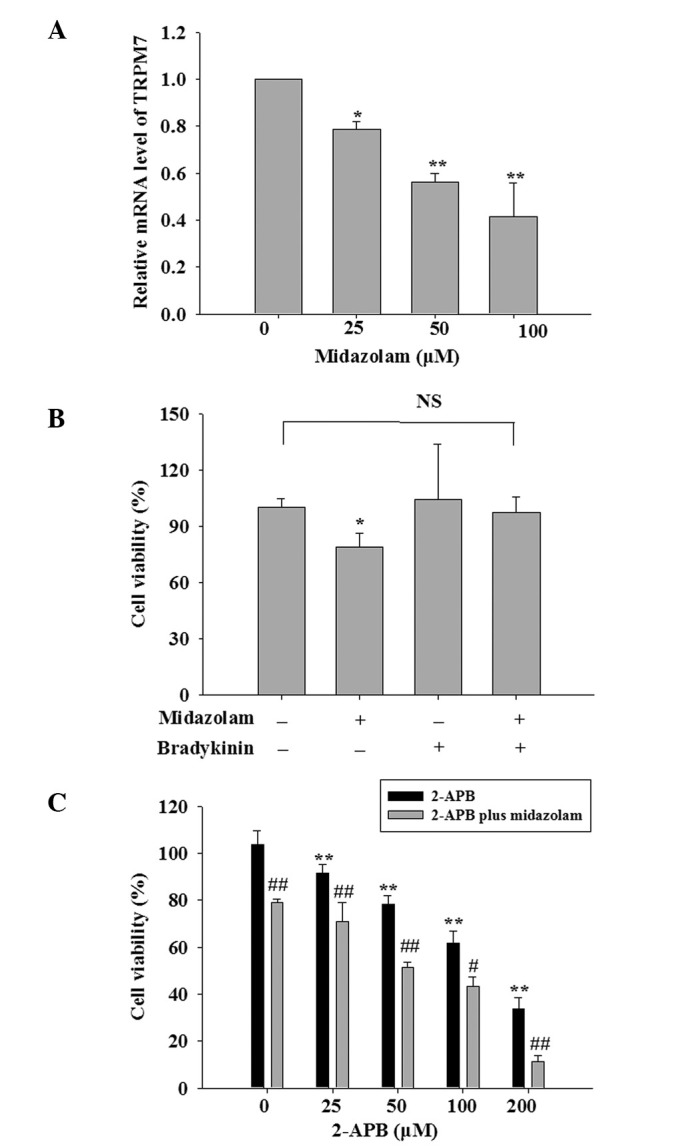
Anti-proliferative activity of midazolam was mediated by suppression of TRPM7. (A) Midazolam repressed the transcriptional expression of TRPM7 dose-dependently. FaDu cells were treated with 0 (control), 25, 50 and 100 *μ*M midazolam for 48 h and then subjected to qPCR. (B) Bradykinin, a specific TRPM7 activator, reversed the proliferation inhibition by midazolam. (C) 2-APB, a non-specific TRPM7 inhibitor also inhibited cell proliferation and enhanced the effect of midazolam. FaDu cells were treated with 50 *μ*M midazolam and co-cultured with bradykinin (200 *μ*M) or 2-APB (25, 50, 100 and 200 *μ*M) for 48 h. The data are the mean ± SD (n=3), ^*^P<0.05, ^**^P<0.01, compared with the control and ^#^P<0.05, ^##^P<0.01, compared with the group treated with 2-APB. TRPM7, transient receptor potential melastatin 7; qPCR, quantitative real-time PCR.

Based on the evidence that TRPM7 exists in FaDu cells and silencing the TRPM7 channel inhibits cell proliferation (5), it was investigated whether the anti-proliferative activity of midazolam is mediated by the inhibition of TRPM7 expression. As shown in Fig. 5A, the data from the RT-qPCR analysis showed that the addition of 25, 50 and 100 *μ*M midazolam to the medium decreased the mRNA level of TRPM7 by 21.5, 43.8 and 58.7%, respectively. Subsequently, bradykinin, a TRPM7 channel activator, was used to examine whether the activation of TRPM7 abrogates the inhibition of proliferation by midazolam in FaDu cells. Fig. 5B shows that the addition of 200 *μ*M bradykinin with 50 *μ*M midazolam in the culture medium reversed the inhibition of cell growth by midazolam, suggesting that TRPM7 inhibition contributes to the inhibitory effect of midazolam on cell growth and proliferation. Furthermore, the effect of 2-APB, a non-specific TRPM7 inhibitor, on the proliferation of FaDu cells was evaluated. As shown in Fig. 5C, 2-APB also inhibited the proliferation of FaDu cells and the combined effect of 2-APB and midazolam appeared to be additive for inhibiting cell growth.

Taken together, these results indicate that the anti-proliferative activity of midazolam was independent of the classic BR pathway but dependent on TRPM7 inhibition.

### Diazepam and clonazepam also exhibit inhibitory activities on the proliferation of FaDu cells

**Figure 6:**
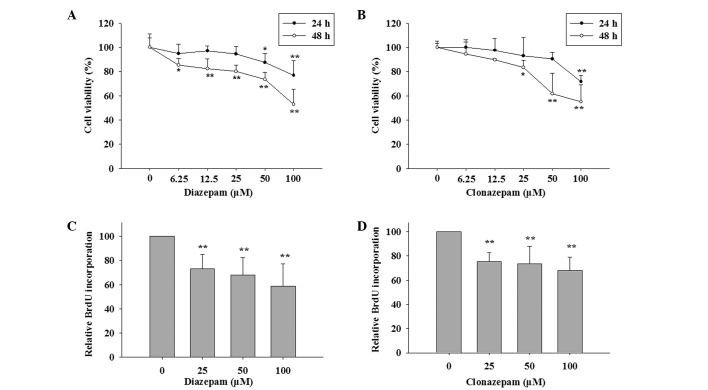
Diazepam and clonazepam also induced proliferation loss in FaDu cells. (A) Time and (B) dose-dependent effects of diazepam and clonazepam on cell viability according to MTT assays. FaDu cells were incubated with 0 (control), 6.25, 12.5, 25, 50 and 100 *μ*M midazolam for 24 and 48 h. The data are the mean ± SD (n=5), ^*^P<0.05, ^**^P<0.01 compared with the control. Effects of (C) diazepam and (D) clonazepam on cell proliferation according to BrdU incorporation assays. FaDu cells were incubated with 0, 25, 50 and 100 *μ*M midazolam for 48 h. The data are the mean ± SD (n=3), ^*^P<0.05, ^**^P<0.01, compared with the control.

To confirm whether midazolam-induced proliferation loss is unique, the effects of other available BZs, including diazepam and clonazepam, on the cell proliferation of FaDu cells were investigated. Similar to midazolam, diazepam and clonazepam exhibited potent anti-proliferative activities at almost the same concentration range (25–100 *μ*M). Cell survival analysis showed that diazepam exhibited anti-growth activity at 24 h which became more evident at 48 h and cell viability was decreased by 19.9, 26.5 and 47.2% with 25, 50 and 100 *μ*M diazepam, respectively (Fig. 6A). In response to 25, 50 and 100 *μ*M clonazepam, the cell viabilities were 16.7, 38.3 and 44.8%, respectively (Fig. 6B). BrdU incorporation assays also provided evidence that diazepam and clonazepam significantly repressed cell proliferation (Fig. 6C and D).

## Discussion

In cancer cells, TRP ion channels, in combination with Ca^2+^ pumps and exchangers, maintain cellular Ca^2+^ homeostasis by driving the influx of Ca^2+^ across the plasma membrane into the cell (8–10). The ability of TRP channels to regulate [Ca^2+^]i and Ca^2+^-dependent tumorigenic pathways, such as proliferation, suggests that therapies modulating TRP channels in cancer cells may be a therapeutic option. Among the members of the TRP families, the TRP vanilloid (TRPV) 6, TRPM1 and 8 channels are most commonly reported to be associated with malignant cell growth and cancer progression (4,11). An accumulating amount of data shows that TRPM7 also acts as a promoter of proliferation, migration and even carcinogenesis in lung carcinoma, pancreatic carcinoma and breast cancer (12–14). This suggests the potential of TRPM7 as a valuable target for the pharmaceutical intervention of cancer. Evidence demonstrating the presence of TRPM7 in human hypopharyngeal squamous cell carcinoma FaDu cells (5), leads to the present study targeting TRPM7 with BZs. The present study suggests the potential exploitation of the anesthetic drug midazolam as a TRPM7 inhibitor. In FaDu cells, midazolam induced cell cycle arrest and thus proliferation loss by a TRPM inhibition-dependent, but not a BR-dependent mechanism. 2-APB, a non-specific TRPM7 inhibitor was also able to mimic the growth-inhibitory activity of midazolam, further supporting the use of TRPM7 as a therapeutic target for cancer.

Ca^2+^ is a ubiquitous intracellular signal responsible for controlling numerous cellular processes and is particularly important at specific phases of the cell cycle. Ca^2+^ and calmodulin (CaM)-dependent signalling is required for Rb phosphorylation and cell cycle progression from the G_1_ to S phase (15). Ca^2+^ and CaM/CaM kinase (CaMK) mainly affect the cell-cycle components by acting directly on the cyclins, CDKs and/or their small protein inhibitors to regulate the assembly and activation of CDK complexes and eventually affect Rb phosphorylation (15,16). Previous studies have demonstrated that the inhibition of CaMK leads to a decrease in cyclin D1 expression, increase in p27 expression, inhibition of CDK2 and CDK4 and G_1_ arrest (17,18). In the present study, midazolam induced an increase in p27 expression, decrease in phosphorylated Rb and cell cycle arrest at G_0_/G_1_ by acting on a Ca^2+^ transport channel, in accordance with the effects of Ca^2+^/CaM/CaMK signaling inhibitors.

Surgical resection of tumors is a necessary treatment for cancers. However, the perioperative period is the most likely time for the cancer to disseminate and metastasize. This is due to the release of cancerous cells during the surgery and suppression of immune function by stress and anesthesia (19,20). It has been shown that the perioperative anesthesia management affects the outcome of patients undergoing tumor resection (21,22). It has also been shown that certain anesthetics including ketamine, thiopental and halothane suppress natural killer cell activity and promote tumor metastasis (23). Opiates are known to inhibit the function of the human immune system (24) and morphine has been implicated in stimulating human microvascular endothelial cell proliferation and angiogenesis *in vitro* and *in vivo*(25). However, the direct effects of anesthetics on the growth and proliferation of cancer cells have not been elucidated. The present study shows that midazolam, a commonly-used anesthetic drug, inhibits the growth and proliferation of human hypopharyngeal squamous cell carcinoma FaDu cells via the suppression of TRPM7 expression. For solid tumors, surgical excision is generally considered to be the most effective approach for removing tumor tissues and alleviating the symptoms caused by the cell masses. During surgery, anesthesia is essential for painless and safe procedures. Administering anesthetics with tumor suppression properties, such as midazolam, may offer an extra protective benefit during tumor resection.

Similar to midazolam, two other BZ drugs, diazepam and clonazepam, also exhibited potent anti-proliferative activities at the same concentration range, indicating the general anti-proliferative effect of BZs. A number of studies have previously proposed that PBR is involved in the effect of BZs on cell proliferation (26,27). However, the present results showing that the anti-proliferative action of midazolam was not mediated by PBR in combination with the fact that non-PBR agonist clonazepam also inhibited cell proliferation, suggest a lack of correlation between the anti-proliferative activities of these BR ligands and PBR. Furthermore, in fibrosarcoma, rat C6 glioma and mouse neuroblastoma, BZs including diazepam and clonazepam inhibit cell proliferation in a PBR-independent manner (28,29).

In summary, the present results demonstrate that targeting TRPM7 with the anesthetic midazolam inhibits the proliferation of human hypopharyngeal squamous cell carcinoma FaDu cells and this may be counteracted by the TRPM7 agonist bradykinin. The concentration of BZs at which they act as TRPM7 blockers and proliferation inhibitors, may be too high for application in cancer therapy. Future studies are likely to concentrate on exploiting more TRPM7 inhibitors and developing BZ derivatives with greater efficacy. Based on the effects of midazolam, bradykinin and 2-APB, it may be concluded that pharmacological modulation of TRPM7 is a promising approach for preventing the growth and proliferation of human head and neck tumor cells.
